# Elevated homocysteine level as an indicator for chronic kidney disease in community-dwelling middle-aged and elderly populations in Taiwan: A community-based cross-sectional study

**DOI:** 10.3389/fmed.2022.964101

**Published:** 2022-08-08

**Authors:** Yu-Lin Shih, Chin-Chuan Shih, Jau-Yuan Chen

**Affiliations:** ^1^Department of Family Medicine, Chang-Gung Memorial Hospital, Linkou Branch, Taoyuan City, Taiwan; ^2^General Administrative Department, United Safety Medical Group, New Taipei City, Taiwan; ^3^College of Medicine, Chang Gung University, Taoyuan City, Taiwan

**Keywords:** homocysteine, middle-age and elderly, chronic kidne disease, kidney function, biomarker

## Abstract

**Background:**

Hyperhomocysteinemia is an important factor for endothelial cell damage and a risk factor for cardiovascular diseases. Chronic kidney disease (CKD) is recognized as a leading burden in Taiwan’s healthcare system. This study aimed to investigate the association between homocysteine levels and CKD in middle-aged and elderly adults from a community in northern Taiwan.

**Methods:**

A total of 396 middle-aged and elderly Taiwanese adults were enrolled and completed the health survey. We divided participants according to tertiles of homocysteine levels as first group (homocysteine level ≤ 11.1 μmol/L), second group (homocysteine level 11.2∼14.3 μmol/L), and third group (homocysteine level > 14.3 μmol/L). CKD was defined as estimated glomerular filtration rate (eGFR) < 60 (mL/min/1.73 m^2^) or urine albumin to creatinine ratio > 30 (mg/g). Pearson correlation was calculated between eGFR and other related risk factors after adjustment for age. The risk of CKD in the second and third groups compared to that in the first group was assessed by multivariate logistic regression after adjustment for age, sex, smoking, hypertension (HTN), diabetes mellitus (DM), body mass index (BMI), dyslipidemia, and uric acid. The Youden index and receiver operating characteristic (ROC) curve were calculated for the optimized cutoff value.

**Results:**

Elevated plasma homocysteine levels were more likely to lower the eGFR and increase the prevalence of CKD. Pearson correlation showed a positive correlation between eGFR and high-density lipoprotein cholesterol, while a negative correlation was observed between homocysteine levels, waist circumference, systolic blood pressure, uric acid levels and BMI (all *p* < 0.05). In the logistic regression analysis, the prevalence of CKD increased, as well as the homocysteine level. The odds ratio of CKD under 95% confidence interval was 2.655 (1.284–5.490) for the third group compared with the first group after adjusting for age, sex, smoking, DM, HTN, dyslipidemia, uric acid, and BMI (*p* = 0.008). The area under the ROC curve was 0.662, and a cutoff value of 15.15 μmol/L for the homocysteine level was obtained for detecting subjects with CKD.

**Conclusion:**

Our study findings revealed that elevated homocysteine levels were significantly associated with CKD and could be used as an indicator of CKD among the middle-aged and elderly populations in Taiwan.

## Introduction

Elevated serum homocysteine levels have been considered an important risk factor for atherosclerosis (1). Many studies have also indicated that hyperhomocysteinemia could be a predictor of peripheral arterial disease (2) and coronary artery disease (3). There are many possible mechanisms underlying the relationship between homocysteine levels and atherosclerosis. These mechanisms include reduction in endothelial cell relaxation (4), decreased synthesis of high-density lipoprotein (HDL; 5), and reduction in endothelial cell growth (6). The study has shown that the elevated serum homocysteine level can compromise the peripheral microvascular endothelial function (7) and homocysteine also plays a crucial role in coronary microvascular endothelial dysfunction (8). Further research also revealed that high homocysteine levels indicated the poor prognosis for patients with stable coronary disease (9). Moreover, elevated homocysteine not only has adverse effects on cardiovascular system but also causes cerebrovascular accidents, retinopathy, nephropathy, and other chronic diseases (10). That evidence implied that the microvascular structure is vulnerable under high homocysteine levels. Due to the microvascular structure of the glomerulus, we suspect that homocysteine level also has relationship with chronic kidney disease (CKD). Recent studies have shed light on the relationship between homocysteine levels and CKD (11). According to recent research, homocysteine also impacts endothelial cells in the glomerulus (12). The accumulation of homocysteine eventually damages the glomerular cells and causes glomerular sclerosis (13).

Kidney also plays an important role in plasma amino acid clearance and metabolism (14). The renal function impairment may compromise the clearance and metabolism of plasma homocysteine, although the detailed mechanism is not entirely understood (15). On the other hand, there are many risk factors like BMI that can also impact the serum homocysteine level and those risk factors should be considered during the investigation of homocysteine (16). Our study discloses the relationship between homocysteine and renal function under meticulous study which involved the related risk factors of high homocysteine level.

This cross-sectional study aimed to further explore the association between serum homocysteine levels and CKD in Taiwan. Adequate participants were included and many related risk factors were collected for adjustment. We also propose the optional cutoff point of homocysteine for predicting CKD in the middle-aged and elderly populations in Taiwan. The result of our study provided substantial evidence for medical practices in screening CKD. By treating CKD in advance, patients can have better life quality and the burden of healthcare can be alleviated.

## Materials and methods

### Study design and participants

The participants in this cross-sectional and community-based study were recruited from a health survey project conducted in 2019 in northern Taiwan. The inclusion criteria were as follows: (1) age between 50 and 85 years, (2) ability to complete a questionnaire, (3) living in the community and ability to walk to the clinic, and (4) completed all examinations. The exclusion criteria were as follows: (1) cardiovascular disease in recent 3 months and (2) missing or incomplete data. Finally, 396 subjects were enrolled in this study and were eligible for the analysis. The sample size was determined by the G*power 3.1 software. The sample of this study included 396 subjects, which implied sufficient statistical power. All participants completed a questionnaire that included their medical history and personal data during the health survey. Blood and urine tests were performed for each participant to obtain biochemical data. A total of 396 participants were divided into three groups based on the homocysteine levels. This study was approved by the Institutional Review Board of Linkou Chang Gung Memorial Hospital, and all participants were informed and provided their consent before enrollment.

### Data collection and laboratory measurements

The content of the questionnaire included sex, alcohol drinking status, and current smoking status. Data on hypertension (HTN), diabetes mellitus (DM), CKD, and dyslipidemia. Resting systolic blood pressure (SBP, mm Hg) and diastolic blood pressure (DBP, mm Hg) were measured at least twice. Waist circumference (WC, cm) was measured at the midpoint between the inferior margin of the last rib and the iliac crest in a horizontal plane while the participant was in a standing position. Body mass index (BMI) was calculated as the person’s weight in kilograms divided by the square of height in meters. The following biomedical laboratory parameters were analyzed at the Roche^®^ model lab at Taiwan E&Q Clinical Laboratory: fasting blood sugar (AC sugar) and homocysteine, low-density lipoprotein (LDL-C, mg/dL), HDL-cholesterol (HDL-C; mg/dL), creatinine (mg/dL), triglyceride (TG, mg/dL), alanine transaminase (ALT, U/L), and uric acid (mg/dL) levels. A standardized kit (cobas™ Homocysteine Enzymatic Assay 100 tests) manufactured by Roche Diagnostics GmbH was used for the determination of homocysteine level in our research. Urine albumin to creatinine ratio (urine ACR, mg/g) was calculated as microalbumin (mg/dL) divided by creatinine (mg/dL). Estimated glomerular filtration rate (eGFR) was calculated using the following equation: 186 × (creatinine/88.4) –1.154 × (age) –0.203 × (0.742 if female) using the modification of diet in renal disease equation.

### Assessment of chronic kidney disease and other variables

Chronic kidney disease was defined as the presence of kidney damage (urine ACR ≥ 30 mg/g) or decreased renal function with an eGFR < 60 mL/min/1.73 m2 (31). DM was defined as a fasting plasma glucose level ≥ 126 mg/dL or the use of oral antidiabetic drugs or insulin therapy. HTN was defined as SBP ≥ 140 mm Hg, DBP ≥ 90 mm Hg, or the use of medication treatment for HTN. Dyslipidemia was defined as LDL-C level ≥ 130 mg/dL, HDL-C level < 40 mg/dL in men or <50 mg/dL in women, TG level ≥150 mg/dL, TC level ≥200 mg/dL, or the use of lipid-lowering medication. Drinking status was defined as alcohol drinking frequency ≥ 3 days/week.

### Statistical analysis

Participants were divided into three groups according to the homocysteine level: first group (homocysteine level ≤ 11.1 μmol/L), second group (homocysteine level 11.2∼14.3 μmol/L), and third group (homocysteine level > 14.3 μmol/L). Then, the laboratory and clinical data were analyzed within each group. We checked the normality of the continuous variables by using Shapiro–Wilk Normality Test. We presented data as mean ± [SD] if our data is consistent with a normal distribution variables, and median [Q1, Q3] if the variables (AC sugar, TG level, HDL-C level, ALT level, Creatinine level, and Urine ACR) significantly deviated from a normal distribution in Table 1. *P*-value were derived from one-way ANOVA for data consistent with a normal distribution and Kruskal–Wallis ANOVA for data consistent with non-normal distribution. The categorical variables were analyzed by the chi-square test and expressed as n (%). The correlation between eGFR and age, AC sugar, homocysteine, TG, HDL-C, and LDL-C level, WC, SBP, BMI, and uric acid level were analyzed using Pearson’s correlation coefficient, which was also adjusted for age. A scatterplot is also displayed. The prevalence of CKD in each group is expressed as a bar chart. Furthermore, multiple logistic regression was performed to evaluate the association between groups and CKD after adjusting for age, sex, BMI, smoking, HTN, DM, dyslipidemia, and uric acid level in each group. Throughout our study, *p* < 0.05 was considered statistically significant. Finally, the Youden index and receiver operating characteristic (ROC) curves were used to determine the optimized homocysteine cutoff value to determine CKD risk. All statistical analyses were performed using SPSS for Windows (version 25.0, released 2011, IBM SPSS Statistics, IBM Corp., Armonk, NY, United States).

**TABLE 1:** General characteristics of the study population according to tertiles of homocysteine levels.

	Tertiles of homocysteine levels				
	Total	First ≤11.1	Second 11.2–14.3	Third >14.3	
					
	*n* = 396	*n* = 132	*n* = 130	*n* = 134	*p*-value
Age (year)	63.72 ± 8.76	63.10 ± 8.49	64.28 ± 9.34	63.78 ± 8.45	0.551
Gender, male (%)	164 (41.4)	24 (18.2)	52 (40.0)	88 (65.7)	<0.001
ACsugar (mg/dL)	99.00 [89.00, 118.75]	97.00 [88.25, 111.00]	99.00 [89.00, 118.00]	103.50 [91.00, 126.25]	0.115
TG level (mg/dL)	118.00 [86.00, 165.00]	111.00 [79.25, 167.75]	111.00 [88.00, 153.25]	126.00 [91.50, 170.25]	0.201
LDL-C level (mg/dl)	109.69 ± 33.99	113.27 ± 32.46	108.09 ± 35.97	107.71 ± 33.46	0.332
HDL-C level (mg/dl)	52.00 [43.00, 61.00]	55.50 [47.00, 65.00]	53.00 [44.00, 64.00]	45.00 [40.75, 56.00]	<0.001
ALT level (U/L)	21.00 [16.00, 30.00]	19.00 [15.00, 26.75]	21.00 [16.00, 34.00]	22.00 [16.75, 32.00]	0.081
Uric acid level (mg/dL)	5.63 ± 1.52	5.13 ± 1.27	5.53 ± 1.35	6.23 ± 1.70	<0.001
Creatinine level (mg/dL)	0.80 [0.68, 0.97]	0.72 [0.61, 0.84]	0.77 [0.69, 0.89]	0.98 [0.83, 1.18]	<0.001
eGFR (mL/min/1.73 m^2^)	87.13 ± 22.40	94.35 ± 20.62	91.33 ± 20.85	75.94 ± 21.31	<0.001
Urine ACR (mg/g)	9.35 [3.80, 24.88]	8.40 [3.90, 15.90]	8.25 [3.30, 22.78]	12.20 [4.78, 48.98]	0.019
CKD, *n* (%)	100 (25.3)	19 (14.4)	27 (20.8)	54 (40.3)	<0.001
Homocysteine level (μmol/L)	13.60 ± 4.90	9.37 ± 1.26	12.66 ± 0.90	18.69 ± 4.92	<0.001
Waist circumference (cm)	85.36 ± 10.83	81.01 ± 10.06	86.01 ± 10.55	89.00 ± 10.40	<0.001
SBP (mm Hg)	137.30 ± 17.49	134.28 ± 16.60	137.29 ± 18.17	140.29 ± 17.29	0.019
DBP (mm Hg)	85.19 ± 10.98	83.61 ± 10.62	84.04 ± 10.35	87.86 ± 11.49	0.002
BMI (kg/m^2^)	25.59 ± 3.84	24.53 ± 3.38	25.49 ± 3.54	26.74 ± 4.23	<0.001
Dyslipidemia, *n* (%)	153 (38.6)	49 (37.1)	43 (33.1)	61 (45.5)	0.105
Current smoker, *n* (%)	50 (12.6)	7 (5.3)	14 (10.8)	29 (21.6)	<0.001
Alcohol drinking, *n* (%)	28 (7.1)	8 (6.1)	9 (6.9)	11 (8.2)	0.789
HTN, *n* (%)	201 (50.8)	55 (41.7)	63 (48.5)	83 (61.9)	0.003
DM, *n* (%)	133 (33.6)	29 (22.0)	49 (37.7)	55 (41.0)	0.002

Continuous variables are expressed as mean ± [SD] if data is consistent with a normal distribution variables, and median [Q1, Q3] if the variables (AC sugar, TG level, HDL-C level, ALT level, Creatinine level, and Urine ACR) significantly deviated from a normal distribution. *P*-value of continuous variables were derived from one-way ANOVA for data consistent with a normal distribution and Kruskal–Wallis ANOVA for data consistent with non-normal distribution.

Categorical variables are expressed as *n* (%) and *p*-values of categorical variables were derived from the chi-square test.

Abbreviation: TG, triglyceride; LDL-C, low-density lipoprotein cholesterol; VLDL-C, very-low-density lipoprotein cholesterol; HDL-C, high-density lipoprotein cholesterol; ALT, alanine aminotransferase; eGFR, estimated glomerular filtration rate; ACR, albumin to creatinine ratio; CKD, chronic kidney disease; SBP, systolic blood pressure; DBP, diastolic blood pressure; BMI, body mass index; HTN, hypertension; and DM, diabetes mellitus.

## Results

A total of 396 middle-aged and elderly individuals from communities in northern Taiwan were enrolled in this study. The participants included 164 men (41.4%) and 232 women (58.6%), with a mean age of 63.72 ± 8.76 years. Table 1 summarizes the demographic and clinical characteristics of the study participants. The enrolled participants were categorized into three subgroups according to their homocysteine levels: first group (homocysteine level ≤ 11.1 μmol/L), second group (homocysteine level 11.2∼14.3 μmol/L), and third group (homocysteine level > 14.3 μmol/L). There were no statistically significant differences in age (*p* = 0.551), AC sugar (*p* = 0.115), TG level (*p* = 0.201), LDL-C level (*p* = 0.332), ALT levels (*p* = 0.081), dyslipidemia (*p* = 0.105), and alcohol consumption (*p* = 0.789) between the first, second, and third groups. The participants in the high homocysteine level group tended to be male (*p* < 0.001) and had higher uric acid level (*p* < 0.001), creatinine level (*p* < 0.001), urine ACR (*p* = 0.007), CKD prevalence (*p* < 0.001), WC (*p* < 0.001), SBP (*p* = 0.019), DBP (*p* = 0.002), BMI (*p* < 0.001), smoking rate (*p* < 0.001), HTN prevalence (*p* = 0.003), and DM prevalence (*p* = 0.002) than those in the low homocysteine level group. Conversely, the participants in the high homocysteine level group had lower HDL-C level (*p* < 0.001) and eGFR (*p* < 0.001) than those in the low homocysteine level group.

**FIGURE 1:**
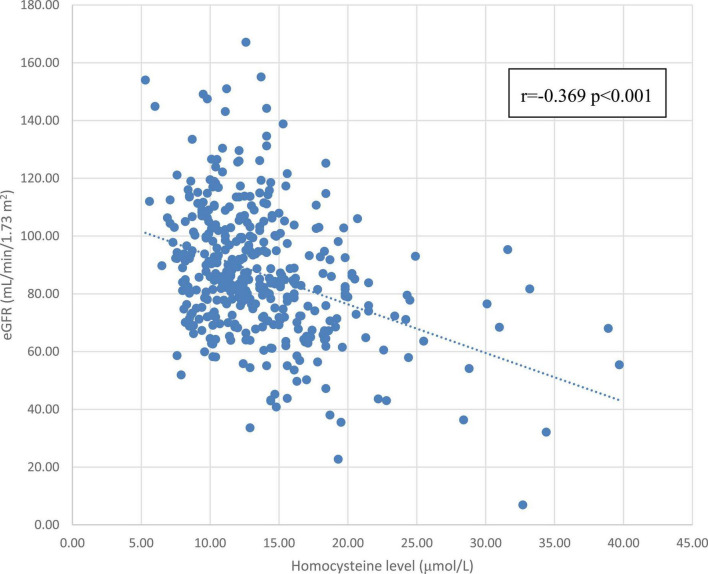
The correlation between the homocysteine level and eGFR. Abbreviations: eGFR, estimated glomerular filtration rate.

Table 2 shows the correlation between eGFR levels and various cardiovascular risk factors. eGFR was positively correlated with HDL-C level (*r* = 0.107, *p* = 0.033) and was negatively correlated with age (*r* = –0.387, *p* < 0.001), homocysteine level (*r* = –0.369, *p* < 0.001), WC (*r* = –0.136, *p* = 0.007), SBP (*r* = –0.165, *p* < 0.001), BMI (*r* = –0.115, *p* = 0.023), and uric acid level (*r* = –0.346, *p* < 0.001). Even after adjusting for age, homocysteine level (*r* = –0.363, *p* < 0.001), WC (*r* = –0.125, *p* = 0.013), SBP (*r* = –0.090, *p* = 0.074), BMI (*r* = –0.149, *p* = 0.003), and uric acid level (*r* = –0.347, *p* < 0.001) still remained statistically significant, except for SBP. Meanwhile, eGFR still remained positive in correlation with HDL-C (*r* = 0.131, *p* = 0.009) after adjusting for age. Figure 1 shows a scatterplot of the eGFR with homocysteine level. Pearson’s correlation was –0.369 with *p* < 0.001.

**TABLE 2:** The correlation between eGFR and metabolic risk factors.

	eGFR (mL/min/1.73 m^2^)			
	Unadjusted		Adjusted for age	
Variable	Correlation coefficient, *r*	*p*-value	Correlation coefficient, *r*	*p*-value
Age (year)	–0.387	<0.001	NA	NA
AC sugar level (mg/dL)	–0.064	0.201	–0.078	0.120
Homocysteine level (μmol/L)	–0.369	<0.001	–0.363	<0.001
TGlevel (mg/dL)	0.066	0.190	0.034	0.502
HDL-C level (mg/dL)	0.107	0.033	0.131	0.009
LDL-C level (mg/dL)	0.098	0.051	0.020	0.698
Waist circumference (cm)	–0.136	0.007	–0.125	0.013
SBP (mm Hg)	–0.165	<0.001	–0.090	0.074
BMI (kg/m^2^)	–0.115	0.023	–0.149	0.003
Uric acid level (mg/dL)	–0.346	<0.001	–0.367	<0.001

Abbreviations: eGFR, estimated glomerular filtration rate; ALT, alanine aminotransferase; TG, triglyceride; HDL-C, high-density lipoprotein cholesterol; LDL-C, low-density lipoprotein cholesterol; SBP, systolic blood pressure; BMI, body mass index; and NA, not applicable.

**FIGURE 2:**
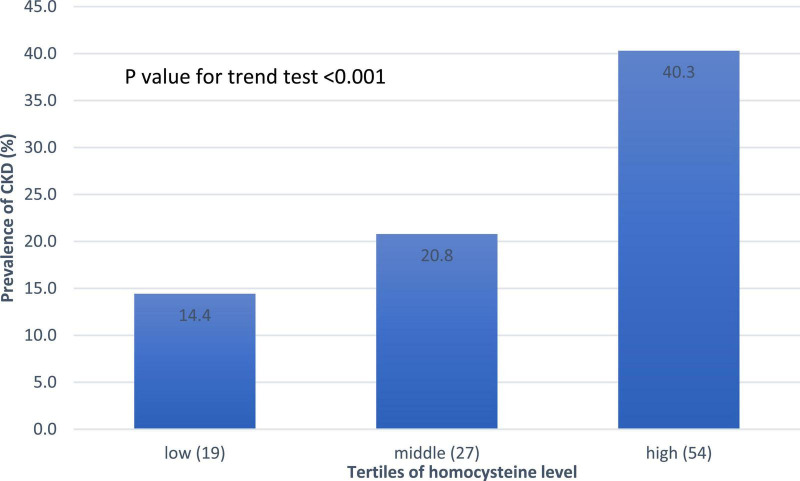
Prevalence of CKD based on the homocysteine level. A linear increasing trend across homocysteine tertiles. Abbreviations: CKD, chronic kidney disease.

**FIGURE 3:**
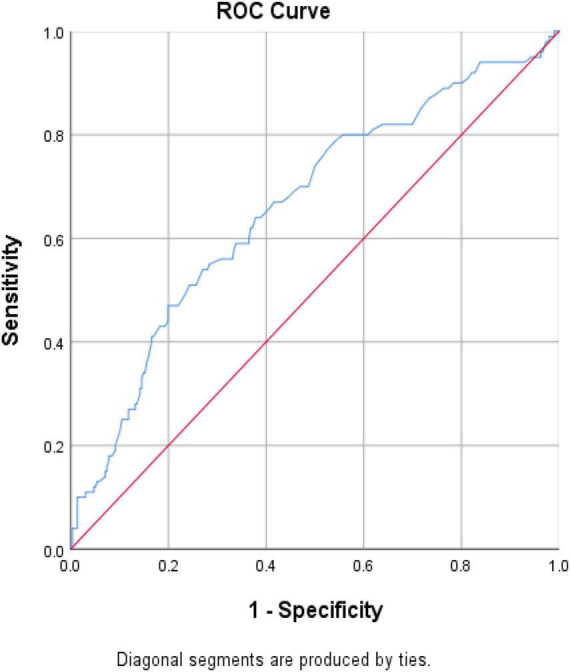
ROC curve for homocysteine level as a biomarker to detect subjects with CKD. Abbreviation: CKD, chronic kidney disease; ROC, receiver operating characteristic.

The association between CKD and homocysteine levels is discussed further. The bar chart in Figure 2 reveals that the third group had 40.3% CKD prevalence, second group had 20.8% CKD prevalence, and first group had 14.4% CKD prevalence. This result showed that high homocysteine level group had a higher CKD prevalence than the low homocysteine level group with statistical significance (*p* < 0.001). In Table 3, a multiple logistic regression model was used to calculate the odds ratio (OR) of homocysteine levels with CKD after adjusting for other risk factors. The third and second groups were compared with the first group in all three models. Model 1 was unadjusted. Model 2 was adjusted for age and sex. Model 3 was adjusted for age, sex, BMI, smoking, HTN, DM, dyslipidemia, and uric acid levels. All ORs decreased and *p*-values increased after adjusting for more factors in the logistic regression model. The variables of third group remained statistically significant after adjustment in model 1 (OR 4.014, 95% CI 2.212–7.286, *p* < 0.001), model 2 (adjusted OR 4.476, 95% CI 2.307–8.687, *p* < 0.001), and even model 3 (adjusted OR 2.655, 95% CI 1.284–5.490, *p* = 0.008). Figure 3 shows the ROC curve for homocysteine levels as a biomarker of CKD. The area under the ROC curve (AUC) was 0.67; the homocysteine level cutoff value was 15.15 μmol/L with a sensitivity of 0.470 and specificity of 0.801 (Table 4).

**TABLE 3:** Multivariate logistic regression analysis of the association between the homocysteine level and CKD according the tertiles of the homocysteine level.

	Model 1			Model 2			Model 3		
Variable	OR	(95% CI)	*p*-value	OR	(95% CI)	*p*-value	OR	(95% CI)	*p*-value
First	1.000	Reference	–	1.000	Reference	–	1.000	Reference	–
Second	1.559	0.818 to 2.971	0.177	1.517	0.777 to 2.962	0.222	1.084	0.524 to 2.240	0.828
Third	4.014	2.212 to 7.286	<0.001	4.476	2.307 to 8.687	<0.001	2.655	1.284 to 5.490	0.008

Model 1: unadjusted.

Model 2: adjusted for age and sex.

Model 3: adjusted for model 2 plus BMI, smoking, HTN, DM, dyslipidemia, uric acid level.

Abbreviation: BMI, body mass index; HTN, hypertension; DM, diabetes mellitus; OR, odds ratio; and CI, confidence interval.

**TABLE 4:** The areas under ROC curve, sensitivity, and specificity of the optimized cutoff points for homocysteine level as a biomarker of CKD.

Variable	Area	*p*-value	Cutoff point	Sensitivity	Specificity
Homocysteine level	0.662 (0.599–0.725)	<0.001	15.15 μmol/L	0.470	0.801

Abbreviation: ROC, receiver operating characteristic; CKD, chronic kidney disease.

## Discussion

Chronic kidney disease is a heavy burden on the elderly and the society worldwide, and more than 750 million people suffer from kidney disease (17). In our community-based research, we investigated the association between homocysteine levels and CKD prevalence in middle-aged and elderly individuals in northern Taiwan. In Table 1, the participants in the high homocysteine level group tended to be male and have a higher uric acid level, creatinine level, urine ACR level, CKD prevalence, WC, SBP, DBP, BMI, smoking rate, DM prevalence, and HTN prevalence than those in the low homocysteine level group, but participants in the high homocysteine level group tended to have lower HDL-C level and eGFR. People with higher homocysteine level were considered to have a higher CVD risk (18), which was predicted by the Framingham Risk Score. According to the Framingham Risk Score, male sex, HTN, smoking status, and SBP were risk factors for CVD, but HDL level was a protective factor against CVD (19). Our results not only corresponded to the relationship between CVD risk factors and the Framingham Risk Score, but were also supported by many previous studies, which indicated a positive relationship between homocysteine level and male sex, HTN, smoking status, and SBP (20–23). The HDL-C level had an inverse relationship with the homocysteine level, which was also noted in a previous study (24). Meanwhile, our research revealed a negative relationship between homocysteine levels and kidney function. In the high homocysteine level group, the uric acid level, creatinine level, urine ACR, and CKD prevalence were all increased, but eGFR was decreased. These findings led us to speculate the relationship between kidney function and homocysteine levels.

In Table 2, we found a negative relationship between eGFR and homocysteine levels, along with other traditional CVD risk factors such as SBP and BMI. After adjusting for age, the negative correlation between homocysteine levels and eGFR still reached statistical significance. Pearson’s correlation between the homocysteine level and eGFR was –0.363 with *p* < 0.001 after adjusting for age, which corresponded to the results of previous studies showing that the homocysteine level was negatively correlated to eGFR and positively correlated to CKD (25, 26). Previous studies and results raise the question of whether the homocysteine level can be an important risk factor for CVD; therefore, we wanted to determine whether a high homocysteine level can be an independent risk factor for CKD.

As shown in Figure 2, the bar chart showed that the high homocysteine level groups tended to have a high CKD prevalence (*p* < 0.001). Furthermore, we analyzed the association between CKD and homocysteine levels using logistic regression with adjustment for other important parameters. In Table 3, the CKD in the second and third groups was compared with the CKD in the first group. The OR for CKD increased in the third group, which always had a greater OR for CKD with statistical significance compared to that in the first group. Even after adjusting for age, sex, BMI, smoking, HTN, DM, dyslipidemia, and uric acid level, the OR for CKD and 95% confidence interval for the third group in comparison with the first group were 2.655 and 1.284 to 5.490, respectively, with *p* = 0.008. This result confirmed that a high homocysteine level (>14.3 μmol/L) was an independent risk factor for CKD. Finally, we used the ROC curve to measure the diagnostic accuracy of the homocysteine level and determine the optimal cutoff value of the homocysteine level for CKD. Figure 3 shows the ROC curve for homocysteine levels as a biomarker to predict CKD. The AUC was 0.662; the homocysteine cutoff value was 15.15 μmol/L with a sensitivity of 0.470 and specificity of 0.801 (Table 4).

Hyperhomocysteinemia is an important biomedical culprit in CKD and is a serious risk factor that ultimately leads to renal failure. Homocysteine is synthesized from methionine as an intermediate product in the methionine cycle, and homocysteine can revert to methionine or cysteine, which plays an important role in cellular redox by modulating the level of glutathione. Homocysteine, cysteine, and glutathione form an interchain or intrachain with hydrogen sulfide between those residues in protein. Hence, homocysteine affects cellular oxidative stress, protein regulation, and cell signaling (27). There are many hypotheses regarding hyperhomocysteinemia-related renal injury.

Homocysteine-induced oxidation causes the primary pathogenesis of hyperhomocysteinemia (28–30). Homocysteine, cysteine, and glutathione are major thiol-containing amino acids in the human body. Thiols are easily catabolized into thiol-thial radicals (RS.), which interact with other thiols to produce disulfide anions (RSSR-), and other oxygen species (ROS), including superoxide anion radicals (O_2_.-) and hydrogen peroxide (H_2_O_2_; 31). Homocysteine can also produce ROS by activating the NADPH oxidase (32, 33). These ROS interrupt normal cellular function and lead to cardiovascular (34) and kidney disease (35). Indeed, studies have shown that hyperhomocysteinemia compromises the microvascular function (7) in many organs with the microvascular system including the kidney (10).

S-adenosyl-L-methionine-dependent methyltransferase (SAM) derived from methionine is catalyzed by SAM-dependent methyltransferases before donating its methyl group. SAM accounts for >90% methylation of nucleic acids, proteins, and lipids. S-adenosylhomocysteine (SAH), which inhibits the methyltransferase reaction, is also a product of SAM-dependent methyl transfer (36). Hyperhomocysteinemia increases intracellular SAH (37) and inhibits SAM-dependent methyl transfer. DNA methyltransferases are vulnerable to high SAH levels. The demethylation of certain genes, including the hTERT gene, affects kidney function (38, 39).

The kidney is the major site of homocysteine metabolism. Most (80%) of the homocysteine binds to plasma proteins, and only the unbound form is subject to glomerular filtration and tubular reabsorption (40, 41). Hyperhomocysteinemia damages renal function as previously mentioned; in return, decreasing renal function leads to further accumulation of homocysteine in CKD (42, 43). In previous studies, they pointed out the positive relationship between homocysteine levels and CKD, but most of them did not consider the influence of other factors and cutoff points for CKD survey. In our study, we considered other traditional CKD factors for adjustment, and the results indicated that a high homocysteine level could be an independent predictor of CKD. In addition to other traditional CKD parameters, homocysteine should be considered an important biomedical marker in healthy screens.

There are several strengths in our study. First, our study has a clear design, sufficient sample size, comprehensive and relevant confounders, and appropriate data analysis. Second, the novelty of our study was discovering the strong relationship between homocysteine and CKD from a community-dwelling population. Third, there was no similar research that investigates this topic in the middle-aged and older populations from the community in Taiwan.

However, there are several limitations to our study. First, the participants in our study were mainly recruited from northern Taiwan. The characteristics of our study subjects may differ from the general population, so the result cannot be generalized to other populations, Second, the number of participants in this study was relatively small, so future studies using a bigger population and random sampling of the community would make the study more convincing. Third, although our study indicated a strong relationship between homocysteine and CKD, this study was a cross-sectional the study, thus, the causal relationship between homocysteine and CKD could not be evaluated and determined. The mechanism of this relationship needs more exploration in the future.

## Conclusion

Our study indicated that a high homocysteine level can be an independent risk factor for CKD in the middle-aged and elderly populations in Taiwan. Further prospective studies should examine and determine if lower homocysteine levels reduce the likelihood of CKD.

## Data availability statement

The raw data supporting the conclusions of this article will be made available by the authors, without undue reservation.

## Ethics statement

The studies involving human participants were reviewed and approved by Institutional Review Board of Linkou Chang Gung Memorial Hospital. The patients/participants provided their written informed consent to participate in this study.

## Author contributions

Y-LS and J-YC composed and conducted the study. C-CS helped data collection. J-YC provided instruction and consultation, project administration, and supervision. All authors contributed to the article and approved the submitted version.

## References

[1] Temple ME Luzier AB Kazierad DJ. Homocysteine as a risk factor for atherosclerosis. *Ann Pharmacother.* (2000) 34:57–65. 10.1345/aph.18457 10669187

[2] Kazemi MB Eshraghian K Omrani GR Lankarani KB Hosseini E. Homocysteine level and coronary artery disease. *Angiology.* (2006) 57:9–14. 10.1177/000331970605700102 16444451

[3] Katsiki N Perez-Martinez P Mikhailidis DP. Homocysteine and non-cardiac vascular disease. *Curr Pharma Design.* (2017) 23:3224–32. 10.2174/1381612823666170317124913 28317478

[4] Zhao J Chen H Liu N Chen J Gu Y Chen J Role of hyperhomocysteinemia and hyperuricemia in pathogenesis of atherosclerosis. *J Stroke Cerebrovasc Dis.* (2017) 26:2695–9. 10.1016/j.jstrokecerebrovasdis.2016.10.012 28986198

[5] Liao D Tan H Hui R Li Z Jiang X Gaubatz J Hyperhomocysteinemia decreases circulating high-density lipoprotein by inhibiting apolipoprotein AI Protein synthesis and enhancing HDL cholesterol clearance. *Circ Res.* (2006) 99:598–606. 10.1161/01.RES.0000242559.42077.22 16931800 PMC4400841

[6] Jamaluddin MDS Chen I Yang F Jiang X Jan M Liu X Homocysteine inhibits endothelial cell growth via DNA hypomethylation of the cyclin a gene. *Blood.* (2007) 110:3648–55. 10.1182/blood-2007-06-096701 17698632 PMC2077313

[7] Toya T Sara JD Lerman B Ahmad A Taher R Godo S Elevated plasma homocysteine levels are associated with impaired peripheral microvascular vasomotor response. *IJC Heart Vasc.* (2020) 28:100515. 10.1016/j.ijcha.2020.100515 32322661 PMC7171522

[8] Ahmad A Corban MT Toya T Sara JD Lerman B Park JY Coronary microvascular endothelial dysfunction in patients with angina and nonobstructive coronary artery disease is associated with elevated serum homocysteine levels. *J Am Heart Assoc.* (2020) 9:e017746. 10.1161/JAHA.120.017746 32993421 PMC7792413

[9] Rallidis LS Kosmas N Rallidi T Pavlakis G Kiouri E Zolindaki M. Homocysteine is an independent predictor of long-term cardiac mortality in patients with stable coronary artery disease in the era of statins. *Coron Artery Dis.* (2020) 31:152–6. 10.1097/MCA.0000000000000800 31609754

[10] Corban MT Lerman LO Lerman A. Ubiquitous yet unseen: Microvascular endothelial dysfunction beyond the heart. *Eur Heart J.* (2018) 39:4098–100. 10.1093/eurheartj/ehy576 30165379

[11] Perna AF Ingrosso D. Homocysteine and chronic kidney disease: An ongoing narrative. *J Nephrol.* (2019) 32:673–5. 10.1007/s40620-019-00622-1 31228166

[12] Yi F Jin S Zhang F Xia M Bao JX Hu J Formation of lipid raft redox signalling platforms in glomerular endothelial cells: An early event of homocysteine-induced glomerular injury. *J Cell Mol Med.* (2009) 13:3303–14. 10.1111/j.1582-4934.2009.00743.x 20196779 PMC3752605

[13] Yi F Li PL. Mechanisms of homocysteine-induced glomerular injury and sclerosis. *Am J Nephrol.* (2008) 28:254–64. 10.1159/000110876 17989498 PMC2820346

[14] Garibotto G Sofia A Saffioti S Bonanni A Mannucci I Verzola D. Amino acid and protein metabolism in the human kidney and in patients with chronic kidney disease. *Clin Nutr.* (2010) 29:424–33. 10.1016/j.clnu.2010.02.005 20207454

[15] Friedman AN Bostom AG Selhub J Levey AS Rosenberg IH. The kidney and homocysteine metabolism. *J Am Soc Nephrol.* (2001) 12:2181–9. 10.1681/ASN.V12102181 11562419

[16] Zaric BL Obradovic M Bajic V Haidara MA Jovanovic M Isenovic ER. Homocysteine and hyperhomocysteinaemia. *Curr Med Chem.* (2019) 26:2948–61. 10.2174/0929867325666180313105949 29532755

[17] Crews DC Bello AK Gamal S. 2019 World kidney day editorial – Burden, access, and disparities in kidney disease. *J Bras Nefrol.* (2019) 41:1–9. 10.1590/2175-8239-jbn-2018-0224 31063178 PMC6534018

[18] Chrysant SG Chrysant GS. The current status of homocysteine as a risk factor for cardiovascular disease: A mini review. *Expert Rev Cardiovasc Ther.* (2018) 16:559–65. 10.1080/14779072.2018.1497974 29979619

[19] Selvarajah S Kaur G Haniff J Cheong KC Hiong TG van der Graaf Y Comparison of the framingham risk score, SCORE and WHO/ISH cardiovascular risk prediction models in an Asian population. *Int J Cardiol.* (2014) 176:211–8. 10.1016/j.ijcard.2014.07.066 25070380

[20] Cohen E Margalit I Shochat T Goldberg E Krause I. Gender differences in homocysteine concentrations, a population-based cross-sectional study. *Nutr Metab Cardiovasc Dis.* (2019) 29:9–14. 10.1016/j.numecd.2018.09.003 30459075

[21] Lin BY Li P Wu XD Li H Zeng ZY. The relationship between homocysteine, blood pressure variability, and left ventricular hypertrophy in patients with essential hypertension: An observational study. *Adv Ther.* (2019) 37:381–9. 10.1007/s12325-019-01154-7 31755036

[22] Awasthi M Omoike OE Paul TK Ridner SL Mamudu HM *An association between smoking status and homocysteine levels and whether this association is modified by sex hormones and cholesterol.* Johnson City, TN: Biomarkers (2019). 10.1080/1354750X.2019.1705395 31835911

[23] Weng H Li Y Fan F Yang H Zhou G Sun P The association between total homocysteine and blood pressure in two independent Chinese populations. *J Hum Hypertens.* (2019) 34:657–65. 10.1038/s41371-019-0288-6 31719670

[24] Momin M Jia J Fan F Li J Dou J Chen D Relationship between plasma homocysteine level and lipid profiles in a community-based Chinese population. *Lipids Health Dis.* (2017) 16:54. 10.1186/s12944-017-0441-6 28288621 PMC5348889

[25] Long Y Nie J. Homocysteine in renal injury. *Kidney Dis.* (2016) 2:80–7. 10.1159/000444900 27536696 PMC4947689

[26] Chao MC Hu SL Hsu HS Davidson LE Lin CH Li CI Serum homocysteine level is positively associated with chronic kidney disease in a Taiwan Chinese population. *J Nephrol.* (2014) 27:299–305. 24430766 10.1007/s40620-013-0037-9

[27] Zhang C Xia M Boini KM Li CX Abais JM Li XX Epithelial-to-mesenchymal transition in podocytes is mediated by the activation of NADPH oxidase in hyperhomocysteinemia. *Pflugers Arch.* (2011) 462:455–67. 10.1007/s00424-011-0981-y 21647593 PMC3299405

[28] Herrmann W Obeid R. Homocysteine: A biomarker in neurodegenerative diseases. *Clin Chem Lab Med.* (2011) 49:435–41. 10.1515/CCLM.2011.084 21388339

[29] Petras M Tatarkova Z Kovalska M Mokra D Dobrota D Lehotsky J Hyperhomocysteinemia as a risk factor for the neuronal system disorders. *J Physiol Pharmacol.* (2014) 65:15–23. 24622826

[30] Yi F Zhang AY Li N Muh RW Fillet M Renert AF Inhibition of ceramide-redox signaling pathway blocks glomerular injury in hyperhomocysteinemic rats. *Kidney Int.* (2006) 70:88–96. 10.1038/sj.ki.5001517 16688115

[31] Jacobsen DW. Hyperhomocysteinemia and oxidative stress: Time for a reality check? *Arterioscler Thromb Vasc Biol.* (2000) 20:1182–4. 10.1161/01.ATV.20.5.1182 10807730

[32] Sipkens JA Hahn N van den Brand CS Meischl C Cillessen SA Smith DE Homocysteine-induced apoptosis in endothelial cells coincides with nuclear NOX2 and nuclear NOX4 activity. *Cell Biochem Biophys.* (2013) 67:341–52. 10.1007/s12013-011-9297-y 22038300 PMC3825580

[33] Ostrakhovitch EA Tabibzadeh S. Homocysteine in chronic kidney disease. *Adv Clin Chem.* (2015) 72:77–106. 10.1016/bs.acc.2015.07.002 26471081

[34] Djuric D Vladimir J Vladimir Z Ivan S. Homocysteine and homocysteine-related compounds: An overview of the roles in the pathology of the cardiovascular and nervous systems. *Can J Physiol Pharmacol.* (2018) 96:991–1003. 10.1139/cjpp-2018-0112 30130426

[35] Shah SV. The role of reactive oxygen metabolites in glomerular disease. *Annu Rev Physiol.* (1995) 57:245–62. 10.1146/annurev.ph.57.030195.001333 7778867

[36] Finkelstein JD. The metabolism of homocysteine: Pathways and regulation. *Eur J Pediatr.* (1998) 157:S40–4. 10.1007/PL00014300 9587024

[37] Perna AF Ingrosso D De Santo NG Galletti P Zappia V. Mechanism of erythrocyte accumulation of methylation inhibitor S-adenosylhomocysteine in uremia. *Kidney Int.* (1995) 47:247–53. 10.1038/ki.1995.31 7731153

[38] Zhang D Sun X Liu J Xie X Cui W Zhu Y. Homocysteine accelerates senescence of endothelial cells via DNA hypomethylation of human telomerase reverse transcriptase. *Arterioscler Thromb Vasc Biol.* (2015) 35:71–8. 10.1161/ATVBAHA.114.303899 25359865

[39] Kloda K Domanski L Kwiatkowska E Borowiecka E Safranow K Drozd A hTERT, BICD1, and chromosome 18 polymorphisms associated with telomere length affect kidney allograft function after transplantation. *Kidney Blood Press Res.* (2015) 40:111–20. 10.1159/000368487 25792135

[40] Selhub J. Homocysteine metabolism. *Annu Rev Nutr.* (1999) 19:217–46. 10.1146/annurev.nutr.19.1.217 10448523

[41] Dudman NP Guo XW Gordon RB Dawson PA Wilcken DE. Human homocysteine catabolism: Three major pathways and their relevance to the development of arterial occlusive disease. *J Nutr.* (1996) 126:1295S–300S. 10.1093/jn/126.suppl_4.1295S 8642474

[42] Rodriguez WE Sen U Tyagi N Kumar M Carneal G Aggrawal D PPAR gamma agonist normalizes glomerular filtration rate, tissue levels of homocysteine, and attenuates endothelial-myocyte uncoupling in alloxan-induced diabetic mice. *Int J Biol Sci.* (2008) 4:236–44. 10.7150/ijbs.4.236 18690293 PMC2500152

[43] Sen U Rodriguez WE Tyagi N Kumar M Kundu S Tyagi SC. Ciglitazone, a PPARgamma agonist, ameliorates diabetic nephropathy in part through homocysteine clearance. *Am J Physiol Endocrinol Metab.* (2008) 295:E1205–12. 10.1152/ajpendo.90534.2008 18780770 PMC2584817

